# Can Ki-67 Play a Role in Prediction of Breast Cancer Patients' Response to Neoadjuvant Chemotherapy?

**DOI:** 10.1155/2014/628217

**Published:** 2014-03-25

**Authors:** Juhasz-Böss Ingolf, Mavrova Russalina, Moga Simona, Radosa Julia, Schmidt Gilda, Rainer M. Bohle, Hasenfus Andrea, Solomayer Erich, Herr Daniel

**Affiliations:** ^1^Department of Obstetrics and Gynecology, Homburg University Medical Centre, 66421 Homburg, Germany; ^2^Department of Pathology, Homburg University Medical Centre, 66421 Homburg, Germany

## Abstract

*Background*. Currently the choice of breast cancer therapy is based on prognostic factors. The proliferation marker Ki-67 is used increasingly to determine the method of therapy. The current study analyses the predictive value of Ki-67 in foreseeing breast cancer patients' responses to neoadjuvant chemotherapy. *Methods*. This study includes patients with invasive breast cancer treated between 2008 and 2013. The clinical response was assessed by correlating Ki-67 to histological examination, mammography, and ultrasonography findings. *Results*. The average Ki-67 value in our patients collectively (*n* = 77) is 34.9 ± 24.6%. The average Ki-67 value is the highest with 37.4 ± 24.0% in patients with a pCR. The Ki-67 values do not differ significantly among the 3 groups: pCR versus partial pathological response versus stable disease/progress (*P* = 0.896). However, Ki-67 values of patients with luminal, Her2 enriched, and basal-like cancers differed significantly from each other. Furthermore, within the group of luminal tumors Ki-67 values of patients with versus without pCR also differed significantly. *Conclusion*. Our data shows that the Ki-67 value predicts the response to neoadjuvant chemotherapy as a function of the molecular subtype, reflecting the daily routine concerning Ki-67 and its impressing potential and limitation as a predictive marker for neoadjuvant chemotherapy response.

## 1. Introduction

Breast cancer is the most diagnosed cancer in women. However, breast cancer mortality rate in industrialised western countries has decreased in the last decades [1, 2]. Early diagnosis and effective therapies contribute greatly to this decrease in mortality rate [3]. Currently the choice of therapy is based on prognostic factors. Different already known prognostic factors such as histological tumour type, tumour size, nodal status, grade, age, and estrogen receptor (ER) status and the proliferation marker Ki-67 influence the type of therapy decision [4]. The clinical use of these factors aims at identifying patients with an unfavourable prognosis and at improving the treatment according to the individual risk (recurrence and mortality). The use of this paradigm over the past three years has led to notable therapy improvement [5, 6].

Moreover, chemotherapy-indication is based on prognostic factors. All patients with an indication for adjuvant chemotherapy can be offered a neoadjuvant treatment [7–10]. A neoadjuvant chemotherapy regiment offers a lot of advantages compared to adjuvant treatment. The response to chemotherapy and therefore also its effectiveness can be better monitored, thus potentially increasing patient compliance. In addition, the use of neoadjuvant cytotoxic treatment may increase the rate of breast conserving therapy and reduce the extent of surgery [8].

Another potential prognostic marker is pathologic complete response (pCR). In many neoadjuvant studies, patients who achieve a pCR showed a better long-term outcome [11, 12]. A pooled analysis of seven randomized trials, including 6,377 patients, showed a significant difference in disease-free survival (DFS) between patients with pCR (ypT0/N0) and patients without pCR. The overall survival (OS) was also better for the former patients. Furthermore, this study shows that pCR is only in highly proliferating breast cancers, like triple negative breast cancer (TNBC), HER2 enriched (HER2 positive plus ER negative), or luminal B/HER2-negative tumours, a good prognostic value, whereas in luminal A and luminal B (ER plus HER2 positive) tumours the pCR is not able to discriminate between good and poor prognosis [13, 14]. In addition to the pCR after neoadjuvant chemotherapy, the proliferation marker Ki-67 is not only a prognostic but also a predictive value.

Ki-67 is a nuclear antigen identified in 1983 which is present in the nuclei of cells in all phases of the cell cycle as well as in mitosis, but quiescent cells in the G_0_ phase do not express it [15–17]. In fact it is the most common marker used in clinical practice. Kwok et al. showed in 2010 that the proliferation marker Ki-67 in needle core biopsy showed better concordance with haematoxylin and eosin mitotic count in surgical excision specimen than routine haematoxylin and eosin mitotic count in needle core biopsy [18]. Relating to neoadjuvant chemotherapy and its response, it has been found that a high level of proliferation activity has predictive value [19]. Fasching et al. showed in 2011 that the response of neoadjuvant chemotherapy in patients with a high Ki-67 level (>30%) was better than in other tumours [20]. Moreover, after a neoadjuvant chemotherapy Ki-67 is still able to function as prognostic marker. Patients with high Ki-67 values in the residual tumour after chemotherapy had a poorer outcome regarding recurrence and mortality. These high risk patients may require further systemic therapy. However, despite these positive qualities Ki-67 is a regular topic of discussion due to its cut-off values and the intra- and interlaboratory reproducibility.

Therefore, the current study was performed in order to analyze retrospectively the predictive value of Ki-67 in prediction of responses of breast cancer patients to neoadjuvant chemotherapy treatment conducted in a German university hospital.

## 2. Patients, Material, and Methods

This retrospective single-center study is composed solely of patients treated by neoadjuvant chemotherapy for invasive breast cancer at a tertiary university center (Saarland University Hospital) between January 2008 and December 2013. The inclusion criteria are that the performance of initial core needle biopsy leading to histopathological diagnosis and surgery following neoadjuvant chemotherapy must be performed at the Department of Gynaecology and Obstetrics of Saarland University Hospital. The exclusion criteria are incomplete data, histopathologic diagnosis and surgery performed at a different institution, and patients with metastasis at the time of initial diagnosis.

Clinical data were obtained using medical records and original pathology reports and collected in an Excel database (Microsoft Corporation, Redmond, WA, USA). The following parameters were assessed: patient's age, tumor size (defined as sonographic diameter (mm) on diagnosis), initial tumor stage and nodal status according to TMN classification, histologic subtype, estrogen receptor status, progesterone status, HER2 status, grading and proliferation status as assessed by Ki-67 staining, neoadjuvant chemotherapy regime and neoadjuvant targeted therapy, posttherapeutic sonographic tumor diameter (mm), posttherapeutic histologic tumor diameter (mm), and posttherapeutic tumor stage and nodal status. Histopathological regression was classified using the semiquantitative scoring system according to Sinn from 0 to 4 (0 = no effect, 1 = resorption and tumor sclerosis, 2 = minimal residual invasive tumor [<0.5 cm], 3 = residual noninvasive tumor only, and 4 = no tumor detectable). A regression grade of four according to Sinn was defined as pathologic complete response (pCR) and a regression grade from two upwards was defined as pathologic partial remission (pPR).

Clinical response was assessed based on a physical examination, mammography, and ultrasonography according to the Response Evaluation Criteria in Solid Tumors (RECIST) [12]. A clinical complete response (cCR) was defined as the disappearance of all known lesions; a clinically partial response (cPR) was defined as a ≥30% reduction in the sum of the longest diameter (LD) of the primary lesion; progressive disease (PD) was defined as a ≥20% increase in the sum of the LD of the primary lesion; and stable disease (SD) was defined as neither sufficient shrinkage to qualify for cPR nor sufficient increase to qualify for PD. The study protocol was approved by the hospital's ethics board and informed consents were obtained from patients in the study.

All histopathological parameters included were derived from the original pathology reports. Tumor tissue was neutral-buffered, formalin-fixed, and paraffin-embedded. Staining of the pretreatment core biopsies was performed using monoclonal rabbit antibodies against estrogen receptor-alpha (clone SP1, DCS Hamburg, Germany), monoclonal rabbit antibody against the progesterone receptor (clone SP2, DCS Hamburg, Germany), and monoclonal antibody against Ki-67 (clone MIB-1, DAKO, Glostrup, Denmark), each according to the manufacturer's instructions using a slide stainer (BenchMark ULTRA, Ventana Medical Systems, Arizona, USA). For evaluation of Ki-67, areas with the highest Ki-67 labeling were investigated. Visualization of antigenic sites was performed using the DakoEnVision kit (Hamburg, Germany). For staining of Her2/neu rabbit antibody was used (A0485, DAKO, Glostrup, Denmark). Her2 status was given on a scale from 0 to 3+. A score of 0 or 1+ was regarded as Her2 negative and a score of 3+ as positive. In case of intermediate score (2+) samples were tested for gene amplification using a Her2 fluorescence in situ hybridization kit (Zytolight, SPEC HER2/CEN17 Dual Color Probe, Zyto Vision Ltd., Bremerhaven, Germany). Hereby gene copy numbers of HER2 and centromeres of the corresponding chromosome 17 were retrieved. A HER2/CEN17 ratio of > 2.2 was considered as amplification of HER2. Cases with a ratio between 1.8 and 2.2 were reevaluated by repeating the staining procedure [12]. Scoring was performed according to standardized protocols by specialized pathologist at the Department of Pathology, Saarland University Hospital.

We only analysed 77 patients in this study, since for the missing cases Ki-67 data was not available. Data was collected in an EXCEL database (Microsoft Corporation, Redmond, WA, USA) and statistical calculations were performed with SPSS (SPSS Inc. Chicago, IL, USA). One way analysis of variance (ANOVA) and paired samples *t*-test were used for analysis. A *P* value <0.05 was considered to indicate statistical significance. Data is reported as mean ± standard error.

## 3. Results

More than 1,000 patients with breast cancer were treated between 2008 and 2013 at the University Hospital of the Saarland. A total of 114 patients received neoadjuvant chemotherapy during this period. The complete medical records, including patient characteristics, tumor characteristics, treatment data, and epidemiological data, and furthermore a Ki-67 determination from 77 patients were analyzed retrospectively. The results of the analysis of these 77 patients are presented in this section.

**Figure 1:**
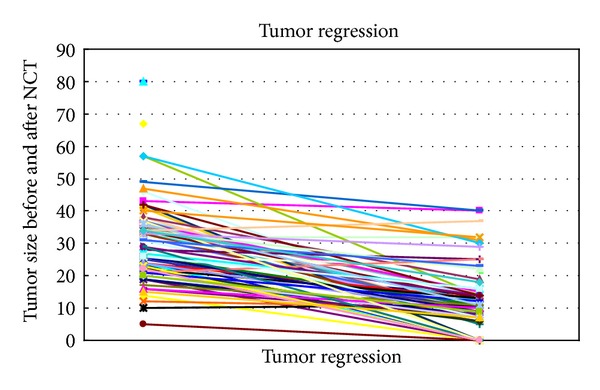
Tumor response, as measured from the maximum initial sonographic diameter and the maximum histological diameter after neoadjuvant chemotherapy and surgery. Data is presented for the values of *n* = 77 patients.

**Figure 2:**
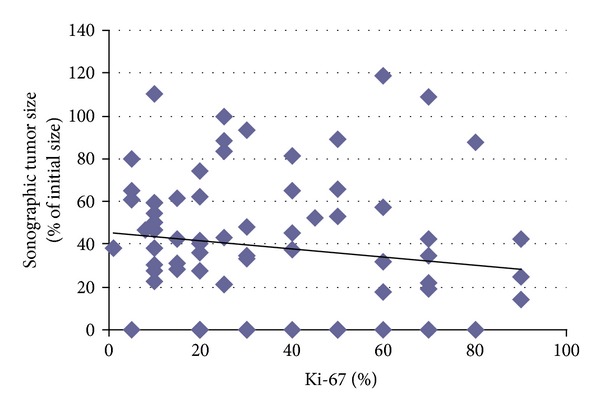
Ki-67 values and sonographic response after neoadjuvant chemotherapy. Tumor sizes are presented in % of the initial sizes.

**Figure 3:**
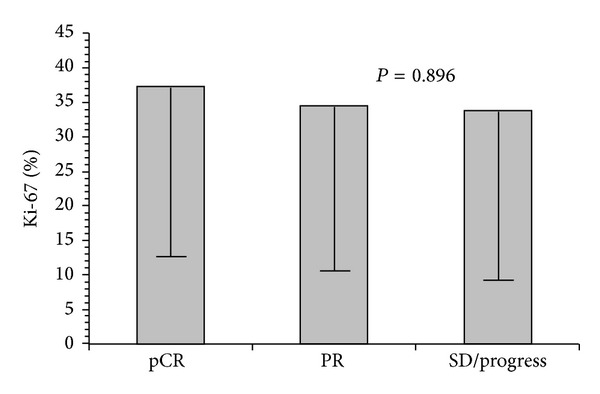
Ki-67 in dependence of the pathological response. pCR = complete pathological response, PR = partial either clinical or pathological response, and SD/progress = stable disease or a progress (mean ± standard deviation).

The average patients' age was 57,8 years old when receiving the initial diagnosis of breast cancer. Tumor characteristics including tumor entity, the initial size of the tumor, the TNM status, the Ki-67 determination, the hormone receptor, and Her2 status were recorded, as shown in Table 1. All patients received neoadjuvant chemotherapy; none received a primary hormone therapy. Information about the administered chemotherapy and in some cases an additional targeted therapy depending on the receptor status is presented in Table 2. Table 2 also includes information about the average tumor size in ultrasound imaging after the neoadjuvant chemotherapy, the average size according to pathological assessment, the TNM status after the treatment, and the pathological response as in Sinn's assessment. The development of tumor size, before the start and after the completion of the neoadjuvant treatment is shown in Figure 1. The average Ki-67 value in our patients collectively was 34,9 ± 24,6% (range 1–90%). A correlation between the Ki-67 value and a response to the neoadjuvant chemotherapy is illustrated in Figure 2. Twenty patients showed a complete pathological response (pCR), thirty-eight patients showed a partial either clinical or pathological response, and seventeen patients had a stable disease or a progress of the disease after finishing the neoadjuvant chemotherapy (Figure 3). In the patient collective with a complete pathological response, the average Ki-67 value was the highest with 37.4 ± 24.0%. Patients with a partial pathological response showed an average Ki-67 value of 34.7 ± 25.5%. Patients with stable disease or rather progress had an average Ki-67 value of 33.8 ± 25.8%. The Ki-67 values do not differ significantly among the 3 groups (*P* = 0.896) as illustrated in Figure 3.

Additionally we divided our patients collectively into 3 groups depending on the cut-off values for Ki-67. We tried to find any differences in initial tumor size and tumor characteristics and investigated the predictive value of Ki-67 for the success of neoadjuvant chemotherapy by correlating it to the pathological response (Table 3). Group A (*n* = 20) represented a group of tumors showing low Ki-67 values (≤15%), group B (*n* = 37) included tumors with an average Ki-67 value between 15 and 50%, and group C (*n* = 18) consisted of tumors with high average Ki-67 values of more than 50%. There were no significant differences between the 3 groups concerning initial tumor diameter, postoperative histologic tumor diameter, change from initial to posttreatment tumor size in ultrasound imaging, pathological assessment, grade of regression according to Sinn, and the number of patients with a complete pathological response (pCR).

However, subdividing the patients according to the molecular subtype of their cancer (Luminal, Her2 enriched, and basal-like), we detected significant differences of Ki-67 between those groups: Ki-67 values of triple negative cancers were 60.4 ± 18.3%, Her-2 positive cancers were 25.4 ± 12.6%, and luminal tumors 22 ± 19.5% (*P* < 0.0001). In addition, within the group of luminal tumors Ki-67 values of patients with versus without pCR differed significantly: patients receiving a pCR presented with Ki-67 values of 50 ± 36.5% versus 18.1 ± 12.9% (*P* = 0.001) (Table 4).

## 4. Discussion

In this retrospective study, 77 breast cancer patients receiving neoadjuvant chemotherapy were analysed concerning Ki-67 and its impact as predictive marker for chemotherapy response. We found a trend towards the highest Ki-67 values in patients achieving a pCR as compared to patients with partial response, stable disease, or progress. However, the observed differences are not significant. In addition, three different groups referring to low, medium, or high levels of Ki-67 have been formed and analysed by correlating the groups to the pathological response. Again no significant differences could be found.

The average Ki-67 value in our population was 34,9% ranging from 1 to 90%. Even in the pCR group with an average Ki-67 of 37,4% the values ranged ±24%. This average value showed a higher trend as compared to the group with partial response, stable disease, or progress. Although the St. Gallen Consensus 2013 recommended the use of Ki-67 as additional factor in order to distinguish the large group of receptor positive breast cancers in luminal A and B, there are several problems concerning the detection of Ki-67. One of the problems of Ki-67 use is the large inter- and intraobserver variation. In 2013 Polley et al. compared the Ki-67 levels in eight of the world's most experienced laboratories and observed a large variation among those laboratories [21]. The most commonly used assay to assess Ki-67 is immunohistochemical (IHC) staining with the MIB-1 antibody. Different groups used different antibodies on paraffin sections after antigen retrieval, such as MM-1, Ki-S5, SP-6, and MIB-1. This might be one reason for the considerable interlaboratory variability. Also, intraobserver variability is a highly discussed issue. The Ki-67 score is defined as the percentage of total number of tumor cells with nuclear staining. Some pathologists estimate the percentage of nuclei staining; others count several hundred nuclei in different areas of tumors to give an overall average index. Therefore more and more automated readers are used. Computer-assisted image analysis can raise the reproducibility of Ki-67 assessment [22], but it has a limited capacity of excluding normal stromal/inflammatory cells [23]. Also, tissue microarray technology has been introduced; its reliability and reproducibility were proven in studies [24]. A standardization of Ki-67 pathological assessment has not yet been accomplished [25]. This lack of consistency across laboratories has thus far limited Ki-67's value. The International Ki-67 in Breast Cancer Working Group was assembled to devise a strategy to harmonize Ki-67 analysis and increase scoring concordance [26].

The above discussed limitations concerning the determination of Ki-67 must be taken into account when discussing our results. However, although the detection and quantification of Ki-67 is difficult our observation of a higher average Ki-67 in the group of patients achieving pCR is in line with several findings of other groups. This concerns also a large number of studies with a neoadjuvant setting. The usefulness of Ki-67 in predicting response and outcome is explored by assessing pretreatment and posttreatment levels of tumor Ki-67 expression in neoadjuvant chemotherapy. Unfortunately, only few of those studies are randomized [27]. Most neoadjuvant chemotherapy studies only perform a univariate analysis looking at response as an outcome [28]. These four studies found Ki-67 to be a predictive marker for either clinical and/or pathological response but only few authors were able to demonstrate Ki-67 as an independent predictor for pCR and overall survival in multivariate models [20].

It has to be declared that a few studies report no correlation between Ki-67 and response to neoadjuvant chemotherapy [29], as we were also not able to find at least a significant correlation between Ki-67 and the response after neoadjuvant chemotherapy. However, since a trend towards response could be observed, it has to be assumed that the lack of significance is due to the relatively small number of patients and the retrospective character of our analysis. In addition, the explanatory power of our data is constricted since we do not provide a multicentric assessment.

In the next step, we subdivided our population into three groups depending on the cut-off levels for Ki-67 (≤15%, 15–50%, and >50%). Again the response after neoadjuvant chemotherapy has been analysed between those groups. However, we were not able to detect any significant differences. This issue addresses another major problem: the varying definition of cut-off values for Ki-67 across the different studies. Klintman et al., for example, uses a Ki-67 cut-off of ≤20%. The St. Gallen Consensus in 2009 classified tumors as low, intermediate, and highly proliferating according to the value of Ki-67 labeling index of ≤15%, 16–30%, and >30% [30]. Fasching et al. and Cheang et al. used a cut-off point for Ki-67 for more than 13% positively stained cells [20]. Denkert et al. in 2013 even pretends that Ki-67 is a significant predictive and prognostic marker over a wide range of cut points, suggesting that data-derived cut point optimisation might not be possible [31]. Nevertheless, Ki-67 may be an important marker regarding the molecular cancer subtypes. We found that Ki-67 values of patients with Luminal, Her2 enriched and basal-like cancers differed significantly from each other. Furthermore, within the group of luminal tumors Ki-67 values of patients with versus without pCR differed significantly. This data is in line with Fasching et al. [20], who found that patients with luminal cancers and pCR have significantly higher Ki-67 values as compared to those without pCR. In our population, patients with triple negative cancer also had the highest levels of Ki-67. However, in contrast to the data of Fasching et al. we did not observe any differences between the group with and without pCR. This might be due to the relatively small subgroup consisting of only 23 patients.

In conclusion, our data shows that the Ki-67 value predicts the response to neoadjuvant chemotherapy in breast cancer patients as a function of the molecular subtype reflecting the daily routine concerning Ki-67 and its impressing chances and yet also its limitations as predictive marker for neoadjuvant chemotherapy response.

## Figures and Tables

**Table 1:** Patient characteristics. Discrete data are given as numbers, continuous as the mean ± standard deviation.

Parameter	Value
Total number of patients	77
Age at first diagnosis (years)	57.8 ± 10.9
Tumor diameter (initial)	31.1 ± 13.6
Histotype	
Invasive ductal	70 (90.9%)
Invasive lobular	6 (7.7%)
Others	1 (1.3%)
Tumor stage (initial)	
1a	—
1b	1 (1.3%)
1c	6 (7.7%)
2	49 (63.6%)
3	5 (6.5%)
4	16 (20.7%)
Nodal status	
Negative	25 (32.4 %)
Positive	52 (67.5%)
Metastasis	
Negative	77 (100%)
Positive	—
Tumor grade	
1	1 (1.3%)
2	37 (48%)
3	39 (50.6%)
Ki-67	34.9 ± 24.6
Estrogen receptor (ER)	
Negative	35 (45.4%)
Positive	42 (54.5%)
Progesterone receptor (PR)	
Negative	40 (51.9%)
Positive	37 (48%)
Her2 receptor	
Negative	57 (74%)
Positive	20 (25.9%)
“Triple negative”	23 (29.8%)

**Table 2:** Therapeutic and postoperative characteristics. Discrete data are given as numbers, continuous as the mean ± standard deviation.

Parameter	Value
Total number of patients	77
Neoadjuvant chemotherapy	
EC/DOC	61 (79.2%)
TAC	11 (14.2%)
Others	5 (6.4%)
Endocrine therapy	—
Neoadjuvant targeted therapy	
Trastuzumab	15 (19.4%)
Lapatinib	3 (3.8%)
Trastuzumab + pertuzumab	2 (2.5%)
Bevacizumab	6 (7.7%)
None	51 (66.2%)
Posttherapeutic sonographic tumor diameter (mm)	12.6 ± 10.0
Postoperative histologic tumor diameter (mm)	15.7 ± 17.1
Postoperative tumor stage	
0	20 (25.9%)
1a	9 (11.6%)
1b	7 (9%)
1c	18 (23.3%)
2	15 (19.4%)
3	6 (7.7%)
4	2 (2.5%)
Grade of regression (Sinn)	
0	3 (3.8%)
1	28 (36.3%)
2	22 (28.5%)
3	2 (2.5%)
4	18 (23.3%)
Unknown	4 (5.1%)

**Table 3:** Therapeutical aspects as a function of Ki-67.

Parameter	Ki-67 ≤15%	Ki-67 16–50%	Ki-67 >50%	*P*
Number of patients	22	37	18	
Initial sonographic tumor diameter (mm)	29.3 ± 14.0	32.2 ± 15.0	31.1 ± 10.4	0.738
Postoperative histologic tumor diameter (mm)	14.6 ± 9.9	18.6 ± 21.7	11.0 ± 15.3	0.312
Change (% from initial tumor size)	57.9 ± 44.9	52.0 ± 66.2	39.4 ± 50.7	0.590
Sonographic change (% from initial tumor size)	47.6 ± 22.2	38.2 ± 32.8	34.4 ± 37.0	0.373
Grade of regression (Sinn)	1.7 ± 1.1	2.2 ± 1.2	2.1 ± 1.4	0.310
Number of patients with pCR	4 (18.1%)	10 (27%)	6 (33.3%)	

**Table 4:** Ki-67 as a function of molecular subtypes.

	Total	pCR yes	pCR no	*P*
Triple negative	*n* = 23	*n* = 5	*n* = 19	*P* = 0.651
	60.4 ± 18.3	57 ± 18.5	61 ± 18.7	
Her2 positive	*n* = 20	*n* = 11	*n* = 9	*P* = 0.576
	25.4 ± 12.6	23.9 ± 10.2	27.2 ± 15.6	
Luminal	*n* = 33	*n* = 4	*n* = 29	*P * ** **=** 0.001**
	22 ± 19.5	50 ± 36.5	18.1 ± 12.9	

	*P * ** **<** 0.0001**			
